# Recoupling of eNOS with Folic Acid Prevents Abdominal Aortic Aneurysm Formation in Angiotensin II-Infused Apolipoprotein E Null Mice

**DOI:** 10.1371/journal.pone.0088899

**Published:** 2014-02-18

**Authors:** Kin Lung Siu, Xiao Niu Miao, Hua Cai

**Affiliations:** Divisions of Molecular Medicine and Cardiology, Departments of Anesthesiology and Medicine, Cardiovascular Research Laboratories, David Geffen School of Medicine at University of California Los Angeles, Los Angeles, California, United States of America; University Heart Center Freiburg, Germany

## Abstract

We have previously shown that eNOS uncoupling mediates abdominal aortic aneurysm (AAA) formation in hph-1 mice. In the present study we examined whether recoupling of eNOS prevents AAA formation in a well-established model of Angiotensin II-infused apolipoprotein E (apoE) null mice by targeting some common pathologies of AAA. Infusion of Ang II resulted in a 92% incidence rate of AAA in the apoE null animals. In a separate group, animals were treated orally with folic acid (FA), which is known to recouple eNOS through augmentation of dihydrofolate reductase (DHFR) function. This resulted in a reduction of AAA rate to 19.5%. Imaging with ultrasound showed that FA markedly inhibited expansion of abdominal aorta. FA also abolished elastin breakdown and macrophage infiltration in the AAA animals. The eNOS uncoupling activity, assessed by L-NAME-sensitive superoxide production, was minimal at baseline but greatly exaggerated with Ang II infusion, which was completely attenuated by FA. This was accompanied by markedly improved tetrahydrobiopterin and nitric oxide bioavailability. Furthermore, the expression and activity of DHFR was decreased in Ang II-infused apoE null mice specifically in the endothelial cells, while FA administration resulted in its recovery. Taken together, these data further establish a significant role of uncoupled eNOS in mediating AAA formation, and a universal efficacy of FA in preventing AAA formation via restoration of DHFR to restore eNOS function.

## Introduction

Abdominal aortic aneurysms (AAA) is a disease that affects the adult population, responsible for more than 70,000 deaths annually in those over the age of 65 [1]. Currently, only larger aneurysms can be treated by surgical correction, which is associated with a 30-day mortality rate of more than 3% [2]. In view of the surgical risk, and lack of medical treatments and prevention strategies for most of the aneurysms, it is important to elucidate detailed molecular mechanisms of the disease to enable new treatment options.

Studies into the underlying mechanisms of this condition show several important processes, such as inflammation, matrix degradation, and remodeling [3]–[6]. As all of these processes are regulated to some degree by reactive oxygen species (ROS), oxidative stress plays an important role in the pathogenesis of AAA in both animal models [7]–[10] and humans [11], [12]. Previous studies have shown that oxidative stress implicated in AAA is often derived from activated vascular smooth muscle cells (VSMCs) and infiltrating macrophages [13]. However, there are some evidences that endothelial cells or endothelial nitric oxide synthase (eNOS) may be involved, and in fact, with uncoupled eNOS serving as a causal factor for AAA development [14]–[17]. Hence, the role that endothelial cells or dysfunctional eNOS plays in the development of AAA warrants further investigation.

In a previous study from our laboratory, we showed that in a strain of mice with a modest deficiency in the eNOS cofactor tetrahydrobiopterin (H_4_B), infusion with angiotensin II (Ang II) for two weeks induces AAA that appears to be among the most severe of available murine models [18]. We further observed that correcting the augmented H_4_B deficiency to restore eNOS function, with oral treatment of folic acid (FA) that restores the expression and activity of the H_4_B salvage enzyme dihydrofolate reductase (DHFR) in the endothelium, eliminated the incidence of AAA [18]. While FA was extremely potent in the prevention of AAA in this model, it is important to examine whether FA targets common pathways of AAA to prevent the disease in other established models of AAA.

In the current study, we examined whether the preventive effect of FA extends to other models of AAA. We chose to use the Ang II infused apoE null model of AAA, as it is a well-characterized murine model for AAA [7], [19], [20] that is different from our previous model of hph-1 mice. The results show that oral treatment of FA significantly reduced the incidence of AAA from 92% to 19.5%. Furthermore, FA treatment significantly improved endothelial specific DHFR expression and activity, restored H_4_B bioavailability, recoupled eNOS, and increased nitric oxide production. Taken together, these data reveal a novel role of eNOS uncoupling in the development of AAA in Ang II-infused apoE null mice, which is exploited by an oral treatment of FA that improves eNOS function to attenuate AAA. These data further confirm a significant role of uncoupled eNOS in mediating AAA formation, and a universal efficacy of FA in preventing AAA formation via restoration of DHFR to restore eNOS function.

## Materials and Methods

### 1) Reagents

Unless otherwise noted, all chemicals were purchased from Sigma-Aldrich at the highest possible purity. Collagenase was obtained from Gibco. Isofluorane was purchased from Piramal Healthcare.

### 2) Animals

All animals and experimental procedures were approved by the Institutional Animal Care and Usage Committee at the University of California, Los Angeles (UCLA). Breeders of apoE null mice were purchased from Jackson Labs (Bar Harbor, ME, Strain B6.129P2-Apoe^tm1Unc^/J), then bred in house for experimental use. Animals were kept in ventilated cages, with free access to water and standard chow, and cared for by the DLAM staff until experiments. Male animals were kept until 6–8 months old before experiments.

### 3) Ang II infusion by osmotic pump

Animals were anesthetized in an isoflurane chamber with 5% isoflurane, and then moved to a nose cone supplying 1.5–2% isoflurane to maintain the anesthetic state. The area on the back between the shoulder blades was cleaned of hair and disinfected. A small incision was made at the cleaned site, and the osmotic pump (Alzet, model 2004) containing Ang II (1000 ng/kg/min) in a delivery solution was inserted into the animal under the skin. The wound site was closed using surgical staples, and the animal was allowed to recover in a heated cage.

### 4) Folic acid treatment

For animal groups treated with folic acid, standard chow was replaced with customerized food tablets containing FA (15 mg/kg/day) [18] two days prior to the osmotic pump implantation, and throughout the study period of 4 weeks of Ang II infusion.

### 5) Tissue collection

Animals were euthanized with CO_2_ 4 weeks after the implantation of osmotic pump containing Ang II. The aorta was rapidly removed from the body, rinsed with ice cold PBS, and cleaned of connective tissue and fat. Determination of the incidence of AAA was made via ultrasound measurements and/or inspection of the abdominal aorta post-mortem.

For histology, small sections (∼2 mm) of abdominal aorta of the suprarenal region was removed and fixed in 10% formalin overnight, followed by another 24 hours in 30% sucrose, then embedded in paraffin. In the case of AAA, a center section of the AAA was prepared. Sections were then sliced at 5 µm.

### 6) Ultrasound imaging of abdominal aorta

Animals were anesthetized with isoflurane, and then placed onto a temperature-controlled table, which also measures ECG for the determination of heart rate. Isoflurane levels were adjusted to maintain heart rate between 400–500 bpm while keeping the animal sufficiently anesthetized (∼1.5–2%). Hair from the abdomen was removed using a hair removal lotion (Veet), and pre-heated ultrasound transmission gel was applied over the cleaned abdomen area. An ultrasound probe (Visualsonics 2100, MS400, 30 MHz) was placed onto the gel to visualize the abdominal aorta transversely. The aorta was confirmed using Doppler mode to detect the presence of pulsatile flow. Consistent localization of image acquisition was insured by visualizing the aorta immediately superior to the branch of the left renal artery in all animals. In the cases of abdominal aneurysms, the measurements were done at the site of maximal aortic diameter.

### 7) Electron spin resonance determination of aortic superoxide production and eNOS uncoupling activity

Aortic superoxide production was determined by electron spin resonance (ESR) as previously described [18]. Briefly, entire freshly isolated aortas were homogenized in lysis buffer (Tris-HCl, NaCl, EDTA, EGTA, Sodium pyrophosphate, β-glycerophosphate, sodium orthovanadate, triton-X) containing 1∶100 protease inhibitor cocktail (Sigma, P8340), centrifuged at 12,000 g for 15 min, and protein supernatant collected. After determination of protein concentration using a protein assay kit (Bio-Rad), 5 µg of protein was loaded into ice-cold and nitrogen bubbled modified Krebs/HEPES buffer (KHB, in mmol/L: NaCl 99; KCl 4.7; MgSO_4_1.2; KH_2_PO_4_ 1.0; CaCl_2_ 1.9; NaHCO_3_ 25; glucose 11.1, NaHEPES 20) containing diethyldithiocarbamic acid (5 µmol/L), deferoxamine (25 µmol/L), and the superoxide specific spin trap methoxycarbonyl-2,2,5,5-tetramethylpyrrolidine (CMH, 500 µmol/L, Alexis). The mixture was loaded into a glass capillary (Kimble, 71900-50), and assayed using electron spin resonance (ESR) spectrophotometer (eScan, Bruker) for superoxide production by taking the difference in the presence or absence of SOD (100 U/mL). To determine eNOS uncoupling activity, measurements were made with the addition of L-NAME (100 µmol/L). The ESR settings used were: biofield, 3494.50; field sweep, 9 G; microwave frequency, 9.75 GHz; microwave power, 21.02 mW; modulation amplitude, 2.47 G; 4096 points of resolution; receiver gain, 1000; and kinetic time, 10 min.

### 8) Electron spin resonance determination of aortic nitric oxide production

Freshly isolated aortic rings from non-aneurismal sections were incubated with freshly prepared nitric oxide specific spin trap Fe^2+^(DETC)_2_ (0.5 mmol/L) in modified Krebs/HEPES buffer at 37°C for 60 min, in the presence of calcium ionophore A23187 (10 µmol/L). After the incubation, the aorta was snap frozen in liquid nitrogen and loaded into a finger Dewar for analysis with ESR spectrophotometer (eScan, Bruker). The instrument settings were as the followings: bio-field, 3,280; field sweep, 77.54 G (1 G = 0.1 mT); microwave frequency, 9.78 GHz; microwave power 40 mW (4 dB); modulation amplitude, 10 G; 4,096 points of resolution; and receiver gain 900.

### 9) HPLC determination of aortic H_4_B content

Entire freshly isolated aortas were lysed in H_4_B lysis buffer (0.1 mol/L phosphoric acid, 1 mmol/L EDTA, 10 mmol/L DL-Dithiothreitol), and then centrifuged at 12,000 g for 3 min. Lysates were then subjected to differential oxidation in acidic (0.2 mol/L trichloroacetic acid with 2.5% I2 and 10% KI) and alkalytic (0.1 mol/L NaOH with 0.9% I2 and 1.5% KI) solutions as described previously [18]. After centrifugation, 10 µL of the supernatant was injected into a HPLC system equipped with a fluorescent detector (Shimadzu). Excitation and emission wavelengths of 350 nm and 450 nm were used to detect H_4_B and its oxidized species.

### 10) Verhoeff-Van Gieson (VVG) staining

Paraffin embedded tissue sections were deparaffinized by sequential washes in xylene (2x), descending alcohol from 100% to 50%, then into distilled water. Sections were then stained in Verhoeff's solution for 70 min, followed by differentiation in 2% ferric chloride for 70 seconds. Sections were then incubated with 5% sodium thiosulfate for 75 seconds, followed by counterstaining with Van Gieson's solution and dehydration with 95% and 100% alcohol, and finally washed in xylene. After drying, the tissues were mounted with Permount (Fisher Scientific).

### 11) Macrophage staining

Tissue sections were deparaffinized and hydrated as described above. After hydration, sections were incubated in methanol containing 1% H_2_O_2_ for 30 min. Sections were then washed with PBST, and blocked in 2% goat serum at RT for 3 hrs prior to being incubated with primary antibody (Mac-3, BD Pharmingen, 550292, 2% in PBS-T) overnight at 4°C. After washing in PBST, sections were incubated with secondary antibody for 1 hr, followed by another wash. Sections were then incubated with the ABC system (goat IgG, Vectastain, Vector Laboratories, Burlingame, CA, 1.5% reagent A and B in PBST) for 30 min, after which the sections were washed in PBST. The Mac-3 staining was visualized by developing with 3,3′-diaminobenzidine (Sigma-Aldrich) for 3 min to achieve the brown coloration. The final sections were dehydrated in ascending grades of ethanol and xylene, and mounted with Permount.

The degree of macrophage infiltration was quantified using Image J by measuring the brightness of mac-3 signals (dark brown) from each vessel segment was measured and normalized by the tissue area.

### 12) Endothelial cell isolation

Endothelial cells were isolated from entire freshly isolated mouse aortas as per our previous study [21]. Briefly, freshly isolated aortas were cut into small pieces (∼2 mm each), and digested in PBS containing collagenase (0.6 mg/mL) for 20 min at 37°C. After digestion, the aortic rings were gently shaken in the buffer to remove endothelial cells from denuded aortas. The endothelial cells in the buffer were pelleted by centrifugation at 1,000 g for 3 min, prior to being subjected to analyses of DHFR expression and activity.

### 13) Western blotting

Western blotting was performed as per standard protocols, using 10% SDS/PAGE gel and transferring to nitrocellulose membrane. Proteins were probed with primary antibodies for DHFR (1∶500, Novus Biologicals, Littleton, CO, H00001719-M01), actin (1∶3000, Sigma-aldrich, A2066), and eNOS (1∶2000, BD transduction laboratories, 610297).

### 14) DHFR Activity assay

DHFR activity was measured via HPLC as previously described [21]. Briefly, cell lysates were incubated with dihydrofolate (50 µmol/L) and NADPH (200 µmol/L) at 37°C for 20 min in a potassium phosphate assay buffer (0.1 mol/L) containing 1 mmol/L DTT, 0.5 mmol/L KCl, 1 mmol/L EDTA, and 20 mmol/L sodium ascorbate at pH 7.4. The reaction was stopped by addition of 0.2 mol/L trichloroacetic acid, and stabilized with a stabilization solution (200 mg sodium ascorbate and 30 mg DTT in 1 mL water, 1∶10 dilution in final solution). The product of this reaction, tetrahydrofolate, was measured using a Shimadzu HPLC system with a C-18 column (Alltech, Deefield) and a fluorescent detection at 295 nm excitation and 365 nm emission. The mobile phase of the HPLC was a 7% acetonitrile with 5 mmol/L KH_2_PO_4_ at pH 2.3.

### 15) Statistical analysis

Differences among different groups of data were compared using unpaired *t*-test for two groups, and ANOVA for multiple groups which was followed by Newman-Keuls post-hoc test. Statistical significance was set at p<0.05. All grouped data are presented as Mean±SEM.

## Results

### 1) Oral FA treatment reduces the incidence of AAA in Ang II-infused apoE null mice

**Figure 1:**
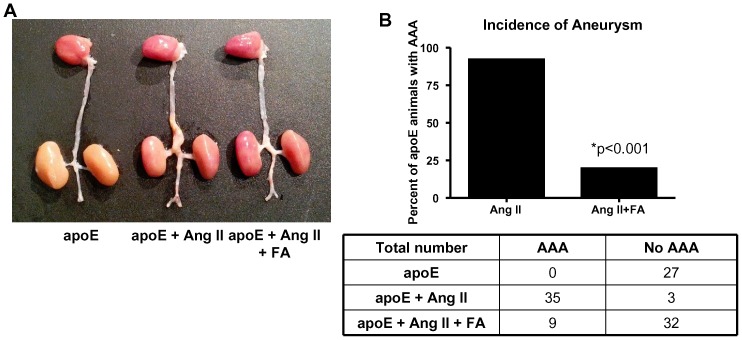
Folic acid greatly reduces the incidence of AAA in Ang II-infused apoE null mice. A) Representative full length aortas from sham operated, Ang II-infused, and Ang II-infused, folic acid (FA) treated apoE null animals. B) The top panel shows the percentage of AAA development in Ang II-infused apoE null mice fed regular chow or chow mixed with FA. The data show that with regular chow, the AAA incidence rate is 92%, while with FA it reduces to 19.5% (*p<0.01). The bottom panel shows the actual number of animals used across all groups: apoE n = 27, apoE+Ang II n = 38, apoE+Ang II+FA n = 41.

The total incidence of AAA across all groups for this study is shown in Figure 1. Data show that after 4 weeks of Ang II-infusion, 92% of the animals developed AAA (n = 38), which is consistent with previous observations from other independent laboratories. Oral FA treatment significantly reduced this incidence to 19.5% (p<0.001, n = 41), indicating that FA is effective in the prevention of AAA in the Ang II-infused apoE null model. None of the animals in this study died from aortic rupture within the study period of 4 weeks.

### 2) Oral FA treatment prevents enlargement of abdominal aorta measured via ultrasound

**Figure 2:**
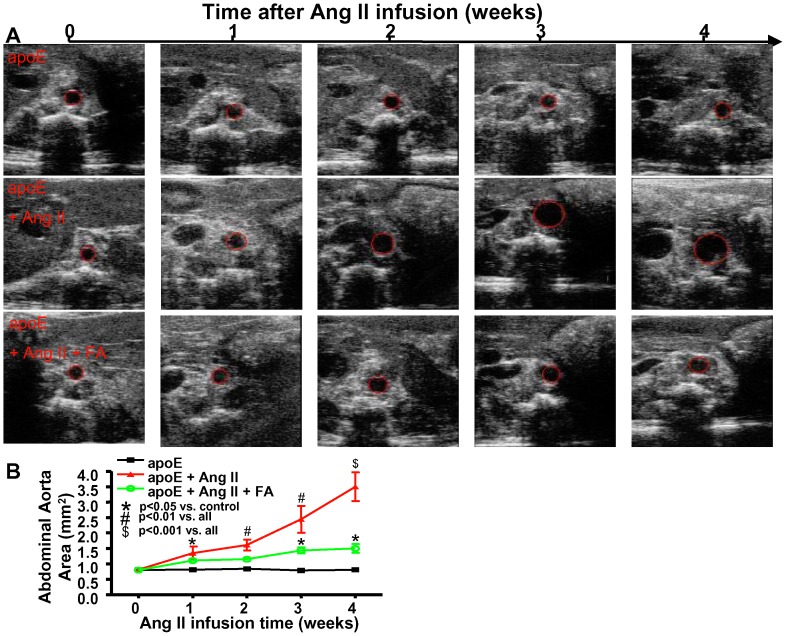
Time course of AAA development and efficacy of folic acid in Ang II-infused apoE null mice measured with ultrasound. A) Representative images of ultrasound taken from sham operated apoE null mice (top row), Ang II-infused apoE null mice with (bottom row) and without (middle row) folic acide (FA) treatment. B) Summarized data from ultrasound measurements of abdominal aorta area. FA significantly reduced abdominal aortic size compared with Ang II alone starting at 2 weeks (n = 4–5 each). * p<0.05 vs. control, # p<0.01 vs. all, $ p<0.001 vs. all.

To assess the time course of AAA development in the Ang II-infused apoE null animals, and the efficacy of FA during this time, we used non-invasive ultrasound to measure the size of the abdominal aorta weekly from a subset of the animals. Figure 2A shows the representative set of images across the 4 weeks, while Figure 2B shows the summarized data (n = 4–5 each). The data show that the abdominal aortas of Ang II-infused apoE null animals were significantly enlarged compared to those of vehicle-infused control animals starting one week after Ang II infusion. FA treatment resulted in marked reduction in aortic size starting from week 2 (Figure 2B).

### 3) Oral FA treatment reduces maladaptive vascular remodeling

**Figure 3:**
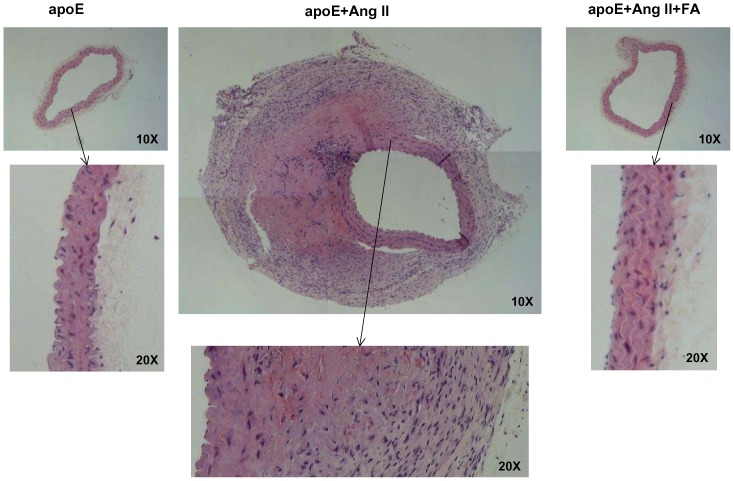
Folic acid reduces adventitial hypertrophy in Ang II-infused apoE null mice. Abdominal aortas were collected from sham operated (left column), Ang II-infused (center column), and Ang II-infused and folic acid (FA, right column) treated apoE null mice 4 weeks after infusion. Tissues were then sectioned and stained for H&E; note partially ruptured aneurysm and attenuation of adventitial hypertrophy by FA.

**Figure 4:**
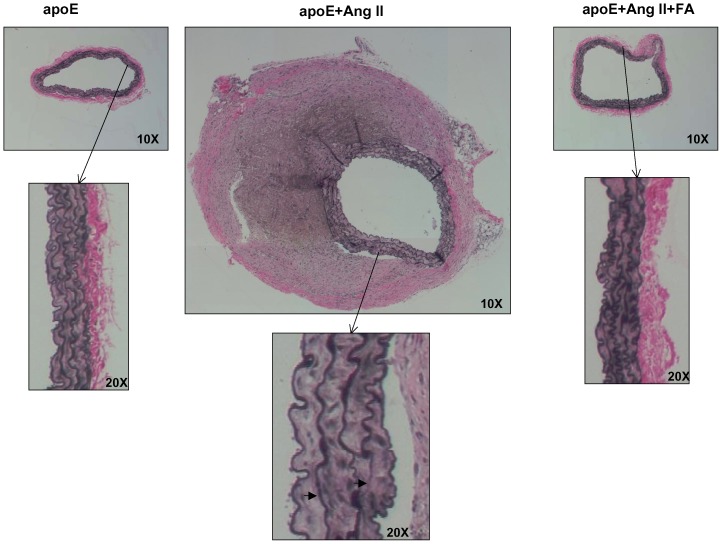
Folic acid reduces matrix degradation in Ang II-infused apoE null mice. Abdominal aortas were collected from sham operated (left column), Ang II-infused (center column), and Ang II-infused and folic acid (FA, right column) treated apoE null mice 4 weeks after infusion. Tissues were then sectioned and stained for VVG; the right arrow in the VVG sections points to a breakdown of elastin fiber, while the left arrow points to a flattening of elastin fiber.

**Figure 5:**
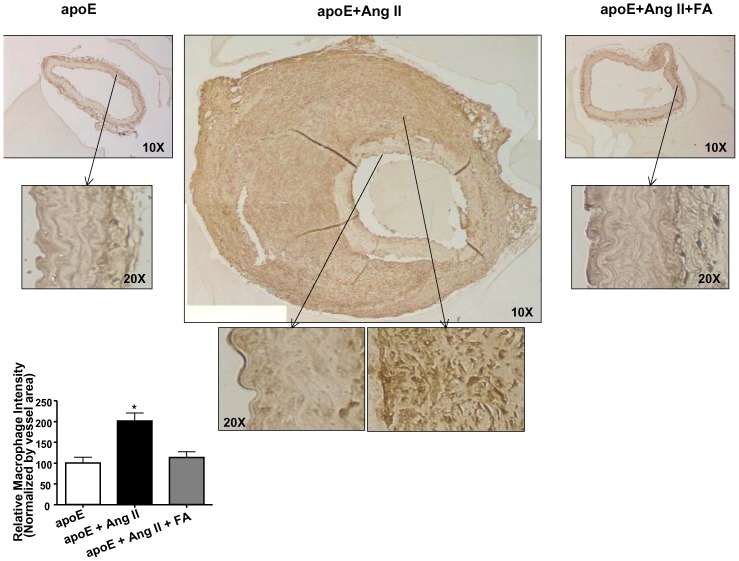
Folic acid reduces inflammation in Ang II-infused apoE null mice. Abdominal aortas were collected from sham operated (left column), Ang II-infused (center column), and Ang II-infused and folic acid (FA, right column) treated apoE null mice 4 weeks after infusion. Tissues were then sectioned and stained for Mac-3 for infiltrating macrophage. Areas within the box were enlarged to better show details of the macrophage infiltration. The degree of macrophage infiltration was measured and quantified in Image J (*p<0.01 vs. all, n = 4 each).

To further analyze the extent of the vascular remodeling that occurred during AAA, we harvested and stained tissue sections of abdominal aortas. Representative images of H&E, VVG, and macrophage staining are shown in Figure 3, 4, and 5. Of note, Ang II infusion induced a marked adventitial hypertrophy (Figure 3). The representative picture also indicated a partially ruptured aneurysm. Furthermore, VVG staining reveals that the elastin fibers were flattened and broken in the Ang II-infused animals (Figure 4), implicating matrix degradation. Treatment with FA largely abolished these responses. Macrophage staining (Figure 5) shows that Ang II infusion significantly increased macrophage infiltration (n = 4 each), while FA treatment was able to attenuate this back to near baseline levels.

### 4) Oral FA treatment reduces total aortic superoxide production, recouples eNOS, improves NO levels, and restores aortic H_4_B bioavailability

**Figure 6:**
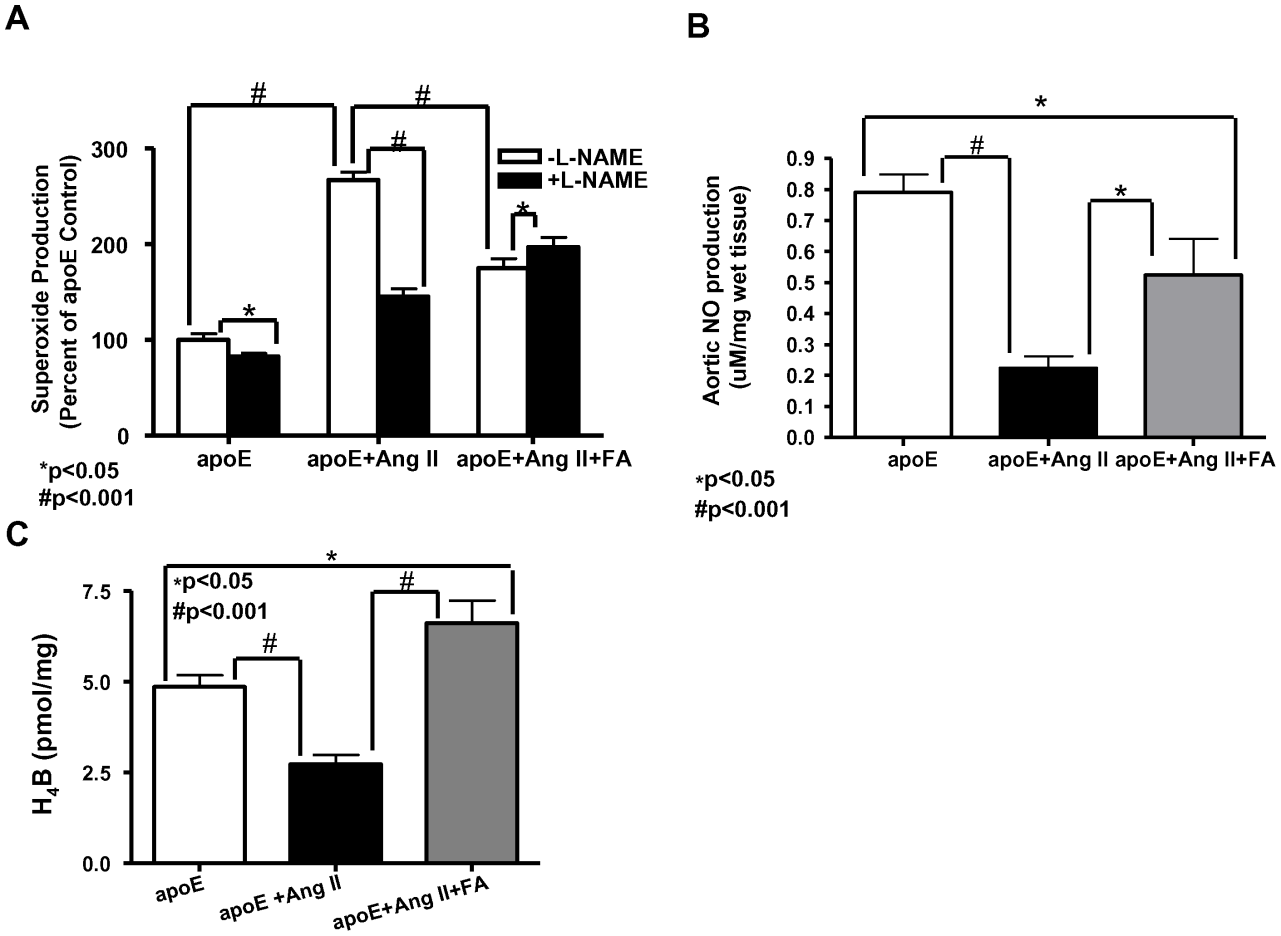
Folic acid reduces superoxide production, restores eNOS function, improves NO production, and increases aortic H_4_B levels in Ang II-infused apoE null mice. A) Total superoxide production measured from aortic homogenates using electron spin resonance (ESR). eNOS uncoupling activity was assessed by comparing measurements with and without the addition of L-NAME, a NOS inhibitor (n = 6). A reduction in superoxide production with L-NAME indicates that NOS is uncoupled and producing superoxide, while an increase in superoxide production with L-NAME indicates that NOS is coupled and producing NO. B) NO production measured from aortas using ESR (n = 5–6). C) Total H_4_B levels measured from aortas using HPLC (n = 5). *p<0.05, #p<0.001.

A previous study by our group showed that eNOS recoupling mediates FA's protective effect against development of AAA [18]. Here, we examined whether the key role that eNOS plays in that model also extends to the model of Ang II-infused apoE null mice. Figure 6A shows the production of superoxide measured from aortic lysates of apoE null animals (n = 6), with and without the presence of the NOS inhibitor L-NAME. The measurements made without L-NAME, shown in white bars, indicate that Ang II infusion significantly increased production of superoxide, while FA treatment reduced this enhanced level.

The measurements made with L-NAME, as shown in the black bars of Figure 4A, are done to assess the coupling state of eNOS. Under normal conditions when eNOS is coupled, the addition of the eNOS inhibitor will increase the measured superoxide production, as eNOS is producing NO to scavenge superoxide. However, when eNOS is uncoupled and producing superoxide, its inhibition will lead to a decrease in measured superoxide. The data show that in the sham operated apoE null animals, there was slight eNOS uncoupling, which was however greatly exacerbated with the infusion of Ang II. FA treatment resulted in re-coupling of eNOS, as shown by the increase in superoxide production with the addition of L-NAME.

Since FA restored eNOS function, we next measured NO levels in isolated aortas from treated apoE null mice. The results, shown in Figure 6B, demonstrate that Ang II infusion significantly decreased the level of NO in the abdominal aortas, while FA treatment significantly increased it (n = 5–6 each).

H_4_B is the essential cofactor of eNOS, while its deficiency is indicative of eNOS uncoupling. Here, we measured H_4_B content from aortas of treated apoE null mice to examine whether FA treatment affects aortic H_4_B content. The data, summarized in Figure 6C (n = 5), show that Ang II infusion significantly reduced aortic H_4_B bioavailability (p<0.01), which was markedly restored by oral FA treatment (p<0.05). Taken together with the previous data, these data show that FA not only reduces superoxide production in the aortas of Ang II-infused apoE null mice, but also recouples eNOS to improve NO levels. This improvement in NO is likely due to an increase in H_4_B levels.

### 5) Oral FA treatment improves DHFR expression and activity in endothelial cells in Ang II-infused apoE aortas

**Figure 7:**
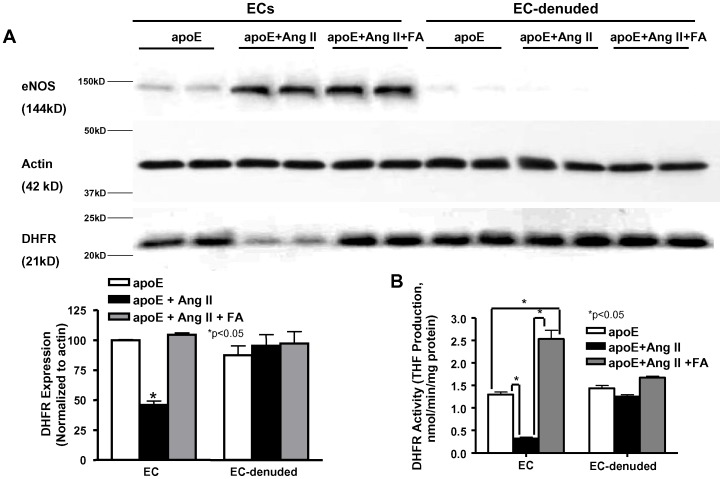
Folic acid treatment restores endothelial specific DHFR expression and activity in Ang II-infused apoE null mice. DHFR expression (A, n = 8–11) and activity (B, n = 5–7) measured from endothelial cells (ECs) and EC-denuded aortas isolated from apoE null mice after 4 weeks of infusion. Data show that DHFR expression and activity was restored in FA treated animals specifically in the endothelial cells. eNOS was stained to show that isolation/removal of ECs from the vessels was successful. *p<0.05.

The above data show that restoration of eNOS coupling, which is tied to the bioavailability of H_4_B, may be important in FA's protection against Ang II induced AAA development in the apoE null animals. Previous studies have shown that FA treatment can recouple eNOS through the improvement of endothelial DHFR function, which is essential in salvaging H_4_B [18], [21]. Here, we examined endothelial DHFR activity and expression in Ang II-infused apoE null mice to test whether DHFR is also improved during FA prevention of AAA in this model. Endothelial cells were isolated from freshly prepared aortas, and Western blots and HPLC were performed to assess DHFR expression and activity respectively on both the isolated ECs and EC-denuded aortas. The top panels of Figure 7A shows the representative Western blots for eNOS (144 kD), actin control (42 kD), and DHFR (21 kD) (n = 8–11 each). eNOS was stained for quality control to examine the effectiveness of the EC isolation/removal. The increase in eNOS expression in the Ang II-infused groups was expected and well documented in previous works [22]–[24]. The summarized data indicate that DHFR was significantly decreased in aortic ECs of Ang II infused apoE null animals, while FA treatment restored this level back to baseline. Similarly, DHFR activity (Figure 7B, n = 5–7) was also markedly reduced in ECs specifically in Ang II infused apoE null mice, but then fully recovered to above baseline levels with oral FA treatment. Of note, DHFR expression and activity remained unchanged in EC-denuded aortas.

## Discussion

In this work, we identified an essential role of endothelial DHFR deficiency and eNOS uncoupling in mediating AAA formation in a well-established AAA model of Ang II infused apoE null mice. This shares similarity with our previous findings establishing causal roles of endothelial DHFR deficiency and eNOS uncoupling in Ang II infused hph-1 mice [18]. Further, we demonstrated that an oral treatment of FA potently attenuates AAA formation in this model from 92% to 19.5%. In addition, Ang II infusion resulted in a large increase in superoxide production, reduced NO production, eNOS uncoupling, and reduction in DHFR expression and activity, all of which were restored by FA treatment. Taken together, these data suggest that FA's protective effects against AAA development are tied to its ability to restore eNOS function. These data further confirm a significant role of uncoupled eNOS in mediating AAA formation and a universal efficacy of FA in preventing AAA formation via restoration of DHFR to restore eNOS function.

In a previous study in our laboratory, we identified a new model for AAA which is characterized by extensive uncoupling of eNOS [18]. In that study, FA was effective in eliminating the incidence of AAA in the animals. Hence, it is important to examine whether eNOS uncoupling plays a role in the development of AAA in other models. In the current study we used the Ang II infused apoE null model of AAA that does not begin with any deficiencies in eNOS cofactor pathways. While it has been reported that apoE null mice do exhibit mild eNOS uncoupling activity at baseline, the general model is a lipid deficiency based model, which is different from our previous study. The results clearly show that FA treatment was protective against AAA, which suggests that FA is likely protective against AAAs in general. In additional experiments we found that FA did not affect lipid profiles during the treatment period.

Oxidative stress has been implicated in the development of AAA. In humans, it was reported that antioxidant systems are inhibited in aneurismal tissue when compared with non-aneurismal sections [11], [12]. However, clinical trials using antioxidants such as vitamin E did not result in any significant benefit in terms of AAA prevention [25], [26]. In this study, while FA did reduce oxidative stress as measured by superoxide production, it is important to note that it also resulted in the restoration of eNOS function. The restoration of eNOS not only reduces superoxide through inhibition of eNOS-derived superoxide production, but also increases NO, which is generally protective in attenuating vascular remodeling. These results match well with our previous study, where the restoration of eNOS was the key in the prevention of AAA development in the hph-1 mice [18]. Taken together, these studies suggest that the restoration of eNOS function could be a possible therapeutic target for the prevention of AAA.

Previous studies using apoE/eNOS double knockout mice demonstrated AAA development when exposed to a Western-type diet for 16 weeks [15], [27], which suggests that eNOS is protective against AAA. In the current study, we used Ang II to induce AAA, which has been shown to result in eNOS uncoupling in this study as well as previous works [21], [24], [28], [29]. Taken together, this suggests that depending upon the coupling state of eNOS, this enzyme can be protective while coupled and producing NO, or causal to the development of AAA while uncoupled and producing superoxide.

One limitation of this study is that for several of the assays, the entire aorta was used, while AAA is localized in the suprarenal region of the aorta. The reason for this is that these assays require more tissue than the size of the AAA. Further, the aneurismal sections sometimes included blood clots in the tissue that could not be removed. The presence of this blood will interfere with the nitric oxide and superoxide measurements. On the other hand, these data seem to suggest that adjacent aortic tissues share similar mechanisms of remodeling, and are indicative of AAA formation in these animals.

In conclusion, this study shows that oral treatment with FA is effective in reducing the incidence of AAA in the Ang II infused apoE null model of AAA. This protective effect can be attributed to an EC specific restoration of the enzyme DHFR, which improves the bioavailability of H_4_B to result in recoupling of eNOS. Taken together with our previous findings from the hph-1 model of AAA, these studies demonstrate that eNOS uncoupling plays an important role in the development of AAA that can be generally observed cross different model systems, and that FA can serve as a universally effective treatment option for prevention and/or treatment of AAA via restoration of DHFR to restore eNOS function.

## References

[1] Centers for Disease Control and Prevention, National Center for Health Statistics. Compressed Mortality File 1999–2010 on CDC WONDER Online Database, released January 2013. Data are compiled from Compressed Mortality File 1999–2010 Series 20 No. 2P, 2013. Available: http://wonder.cdc.gov/cmf-icd10.html. Accessed Jul 13, 2013 12:24:55 PM.

[2] Qadura M, Pervaiz F, Harlock JA, Al-Azzoni A, Farrokhyar F, et al. (2013) Mortality and reintervention following elective abdominal aortic aneurysm repair. J Vasc Surg 57: : 1676–1683 e1671. 10.1016/j.jvs.2013.02.013 23719040

[3] Shimizu K, Mitchell RN, Libby P (2006) Inflammation and cellular immune responses in abdominal aortic aneurysms. Arterioscler Thromb Vasc Biol 26: 987–994. 16497993 10.1161/01.ATV.0000214999.12921.4f

[4] Michel JB, Martin-Ventura JL, Egido J, Sakalihasan N, Treska V, et al (2011) Novel aspects of the pathogenesis of aneurysms of the abdominal aorta in humans. Cardiovasc Res 90: 18–27. 21037321 10.1093/cvr/cvq337 PMC3058728

[5] Tsuruda T, Kato J, Hatakeyama K, Kojima K, Yano M, et al (2008) Adventitial mast cells contribute to pathogenesis in the progression of abdominal aortic aneurysm. Circ Res 102: 1368–1377. 18451339 10.1161/CIRCRESAHA.108.173682

[6] Turner GH, Olzinski AR, Bernard RE, Aravindhan K, Boyle RJ, et al (2009) Assessment of macrophage infiltration in a murine model of abdominal aortic aneurysm. J Magn Reson Imaging 30: 455–460. 19629967 10.1002/jmri.21843

[7] Gavrila D, Li WG, McCormick ML, Thomas M, Daugherty A, et al (2005) Vitamin E inhibits abdominal aortic aneurysm formation in angiotensin II-infused apolipoprotein E-deficient mice. Arterioscler Thromb Vasc Biol 25: 1671–1677. 15933246 10.1161/01.ATV.0000172631.50972.0f PMC3974107

[8] Grigoryants V, Hannawa KK, Pearce CG, Sinha I, Roelofs KJ, et al (2005) Tamoxifen up-regulates catalase production, inhibits vessel wall neutrophil infiltration, and attenuates development of experimental abdominal aortic aneurysms. J Vasc Surg 41: 108–114. 15696052 10.1016/j.jvs.2004.09.033

[9] Thomas M, Gavrila D, McCormick ML, Miller FJ Jr, Daugherty A, et al (2006) Deletion of p47phox attenuates angiotensin II-induced abdominal aortic aneurysm formation in apolipoprotein E-deficient mice. Circulation 114: 404–413. 16864727 10.1161/CIRCULATIONAHA.105.607168 PMC3974117

[10] Xiong W, Mactaggart J, Knispel R, Worth J, Zhu Z, et al (2009) Inhibition of reactive oxygen species attenuates aneurysm formation in a murine model. Atherosclerosis 202: 128–134. 18502427 10.1016/j.atherosclerosis.2008.03.029 PMC2646364

[11] Dubick MA, Hunter GC, Casey SM, Keen CL (1987) Aortic ascorbic acid, trace elements, and superoxide dismutase activity in human aneurysmal and occlusive disease. Proc Soc Exp Biol Med 184: 138–143. 3809168 10.3181/00379727-184-42457

[12] Dubick MA, Keen CL, DiSilvestro RA, Eskelson CD, Ireton J, et al (1999) Antioxidant enzyme activity in human abdominal aortic aneurysmal and occlusive disease. Proc Soc Exp Biol Med 220: 39–45. 9893167 10.1046/j.1525-1373.1999.d01-6.x

[13] Miller FJ Jr, Sharp WJ, Fang X, Oberley LW, Oberley TD, et al (2002) Oxidative stress in human abdominal aortic aneurysms: a potential mediator of aneurysmal remodeling. Arterioscler Thromb Vasc Biol 22: 560–565. 11950691 10.1161/01.atv.0000013778.72404.30

[14] Cafueri G, Parodi F, Pistorio A, Bertolotto M, Ventura F, et al (2012) Endothelial and smooth muscle cells from abdominal aortic aneurysm have increased oxidative stress and telomere attrition. PLoS One 7: e35312. 22514726 10.1371/journal.pone.0035312 PMC3325957

[15] Kuhlencordt PJ, Gyurko R, Han F, Scherrer-Crosbie M, Aretz TH, et al (2001) Accelerated atherosclerosis, aortic aneurysm formation, and ischemic heart disease in apolipoprotein E/endothelial nitric oxide synthase double-knockout mice. Circulation 104: 448–454. 11468208 10.1161/hc2901.091399

[16] Johanning JM, Franklin DP, Han DC, Carey DJ, Elmore JR (2001) Inhibition of inducible nitric oxide synthase limits nitric oxide production and experimental aneurysm expansion. J Vasc Surg 33: 579–586. 11241130 10.1067/mva.2001.111805

[17] Fatini C, Sofi F, Sticchi E, Bolli P, Sestini I, et al (2005) eNOS G894T polymorphism as a mild predisposing factor for abdominal aortic aneurysm. J Vasc Surg 42: 415–419. 16171581 10.1016/j.jvs.2005.05.044

[18] Gao L, Siu KL, Chalupsky K, Nguyen A, Chen P, et al (2011) Role of uncoupled endothelial nitric oxide synthase in abdominal aortic aneurysm formation: treatment with folic acid. Hypertension 59: 158–166. 22083158 10.1161/HYPERTENSIONAHA.111.181644 PMC3668799

[19] Daugherty A, Manning MW, Cassis LA (2000) Angiotensin II promotes atherosclerotic lesions and aneurysms in apolipoprotein E-deficient mice. J Clin Invest 105: 1605–1612. 10841519 10.1172/JCI7818 PMC300846

[20] Satoh K, Nigro P, Matoba T, O'Dell MR, Cui Z, et al (2009) Cyclophilin A enhances vascular oxidative stress and the development of angiotensin II-induced aortic aneurysms. Nat Med 15: 649–656. 19430489 10.1038/nm.1958 PMC2704983

[21] Gao L, Chalupsky K, Stefani E, Cai H (2009) Mechanistic insights into folic acid-dependent vascular protection: dihydrofolate reductase (DHFR)-mediated reduction in oxidant stress in endothelial cells and angiotensin II-infused mice: a novel HPLC-based fluorescent assay for DHFR activity. J Mol Cell Cardiol 47: 752–760. 19660467 10.1016/j.yjmcc.2009.07.025 PMC2784291

[22] Tamarat R, Silvestre JS, Kubis N, Benessiano J, Duriez M, et al (2002) Endothelial nitric oxide synthase lies downstream from angiotensin II-induced angiogenesis in ischemic hindlimb. Hypertension 39: 830–835. 11897773 10.1161/hy0302.104671

[23] Yayama K, Hiyoshi H, Imazu D, Okamoto H (2006) Angiotensin II stimulates endothelial NO synthase phosphorylation in thoracic aorta of mice with abdominal aortic banding via type 2 receptor. Hypertension 48: 958–964. 17000928 10.1161/01.HYP.0000244108.30909.27

[24] Chalupsky K, Cai H (2005) Endothelial dihydrofolate reductase: critical for nitric oxide bioavailability and role in angiotensin II uncoupling of endothelial nitric oxide synthase. Proc Natl Acad Sci U S A 102: 9056–9061. 15941833 10.1073/pnas.0409594102 PMC1157015

[25] The Alpha-Tocopherol Beta Carotene Cancer Prevention Study Group (1994) The effect of vitamin E and beta carotene on the incidence of lung cancer and other cancers in male smokers. The Alpha-Tocopherol, Beta Carotene Cancer Prevention Study Group. N Engl J Med 330: 1029–1035. 8127329 10.1056/NEJM199404143301501

[26] Tornwall ME, Virtamo J, Haukka JK, Albanes D, Huttunen JK (2001) Alpha-tocopherol (vitamin E) and beta-carotene supplementation does not affect the risk for large abdominal aortic aneurysm in a controlled trial. Atherosclerosis 157: 167–173. 11427217 10.1016/s0021-9150(00)00694-8

[27] Chen J, Kuhlencordt PJ, Astern J, Gyurko R, Huang PL (2001) Hypertension does not account for the accelerated atherosclerosis and development of aneurysms in male apolipoprotein e/endothelial nitric oxide synthase double knockout mice. Circulation 104: 2391–2394. 11705813 10.1161/hc4501.099729

[28] Oak JH, Cai H (2007) Attenuation of angiotensin II signaling recouples eNOS and inhibits nonendothelial NOX activity in diabetic mice. Diabetes 56: 118–126. 17192473 10.2337/db06-0288

[29] Dikalova AE, Gongora MC, Harrison DG, Lambeth JD, Dikalov S, et al (2010) Upregulation of Nox1 in vascular smooth muscle leads to impaired endothelium-dependent relaxation via eNOS uncoupling. Am J Physiol Heart Circ Physiol 299: H673–679. 20639222 10.1152/ajpheart.00242.2010 PMC2944492

